# Lower Task-Estimated Visual Working Memory Capacity in Adults with Epilepsy: A Cross-Sectional Quantitative Assessment Using Eye Tracking

**DOI:** 10.3390/diagnostics16193156

**Published:** 2026-09-28

**Authors:** Yulong Li, Ruiqi Yang, Xinyun Xu, Ling Jin, Mingsha Zhang, Lei Chen

**Affiliations:** 1 Department of Neurology, West China Hospital, Sichuan University, No. 28 Dianxin South Street, Chengdu 610041, China; 2 Laboratory of Neuro-Diseases and Multimorbidity, West China Hospital, Sichuan University, Chengdu 610041, China; 3 Engineering Research Center of Brain-Machine Interactive Neuromodulation Sichuan Provincial, Chengdu 610041, China; 4 Sichuan Provincial Model Worker and Craftsman Innovation Studio, Chengdu 610041, China; 5 State Key Laboratory of Cognitive Neuroscience and Learning and IDG/McGovern Institute for Brain Research, Division of Psychology, Beijing Normal University, Beijing 100875, China

**Keywords:** cognitive dysfunction, change localization, change detection, set size, saccades

## Abstract

**Background/Objectives:** Cognitive dysfunction is clinically relevant in epilepsy, and objective task-based measures could complement neuropsychological assessment, but visual working memory across graded loads and task-embedded gaze remain incompletely characterized. We quantified task-estimated capacity and performance using gaze-triggered change localization. **Methods:** We analyzed data from 222 adults with epilepsy, including focal and generalized epilepsy, and 133 healthy controls. Participants identified the changed item in a test array by sustained gaze; set size (2–7) followed a staircase procedure. Outcomes were three task-performance measures, two capacity-related measures, and eight eye-movement measures. Repeated-measure outcomes were analyzed with mixed-effect models and the capacity-related measures by ordinary least squares, with Benjamini–Hochberg correction. **Results:** Adults with epilepsy had lower mean set size and K at set size 4 (K4), lower accuracy, longer response time, and greater final gaze-to-target distance than controls (all *q* < 0.05; absolute Cohen’s *d*, 0.20–0.45). K4 was 2.66 versus 3.03 items. Differences persisted after adjustment for educational attainment and were directionally similar in focal and generalized epilepsy. No interaction between group and set size survived correction for task performance; group differences did not vary detectably across the observed load range. No eye-movement group difference survived correction. **Conclusions:** In a relatively large adult cohort, task-estimated visual working memory capacity and task performance were lower in epilepsy, without broad corrected group differences in the selected array-level eye-movement measures. By combining graded load with gaze-based response registration, the study characterizes the magnitude, load dependence, timing, and spatial precision of visual working memory performance differences within a single task.

## 1. Introduction

Cognitive impairment is common in epilepsy and may be evident at diagnosis, before antiseizure treatment begins [1,2,3]. Its expression is heterogeneous, reflecting etiology, network dysfunction, interictal epileptiform activity, seizures, and treatment [4,5]. This burden is clinically important because seizure-focused assessment alone does not describe how cognition is affected. International recommendations define when neuropsychological assessment should be used in routine epilepsy care [6]. Neuropsychological testing remains the reference approach, while well-defined quantitative tasks may complement it by measuring specific cognitive operations under controlled demands.

Working memory is particularly suitable for such measurement because it supports temporary maintenance and use of information during goal-directed behavior [7,8,9]. Impairment has been reported in pediatric epilepsy [10], genetic generalized epilepsy [11,12], and mixed adult cohorts [13]. Visual working memory offers a tractable framework in which memory load is manipulated within a single task using change-detection or change-localization paradigms [14,15,16,17]. In healthy adults, estimates of the number of items retained typically fall in the range of approximately three to four [18,19,20]. Under explicit assumptions about guessing, fixed-load accuracy can be converted to an item-based K estimate [16,19,21], allowing capacity to be expressed on an interpretable task-derived scale.

Eye movements add a complementary dimension. Gaze influences how objects are sampled, while remembered information can guide subsequent gaze selection [22,23,24,25]. Eye position can also serve as the behavioral response [24,26,27]. With sustained-gaze response registration, the selected location becomes a spatial response measure and verbal or manual output is unnecessary.

Concurrent eye tracking has been used in memory tasks in temporal, mesial temporal, and frontal lobe epilepsy [28,29,30], and in facial emotion recognition in temporal lobe epilepsy [31]. Despite these applications, visual working memory capacity across graded loads remains incompletely characterized in larger adult cohorts spanning focal and generalized epilepsy. It is also unclear whether any performance difference changes with increasing load or is accompanied by broad changes in task-embedded gaze.

We used a gaze-triggered change-localization task with graded set sizes to quantify task-estimated visual working memory capacity and complementary measures of accuracy, response time, and spatial response precision. We also tested whether group differences varied with set size and characterized eye-movement measures during sample and test arrays. By placing these outcomes within one procedure, the study was designed to define the magnitude, load dependence, and gaze-related context of visual working memory performance differences in adults with epilepsy.

## 2. Materials and Methods

### 2.1. Study Participants

Adults with epilepsy were prospectively recruited at West China Hospital, Sichuan University, from November 2023 through August 2024. Eligibility required age 18–60 years and epilepsy meeting the 2014 International League Against Epilepsy practical clinical definition [32]. Exclusion criteria were a seizure within 72 h before assessment; another neurological disorder; cognitive impairment preventing task completion; major ocular disease likely to interfere with eye tracking; or a psychiatric or severe medical condition precluding safe participation. Healthy controls were recruited during the same period and frequency-matched on age, sex, and educational attainment. The same age criterion and the latter four exclusion criteria applied.

The study was approved by the Ethics Committee of West China Hospital, Sichuan University, on 1 November 2023 (No. 2023-2394). Participants provided written informed consent.

### 2.2. Demographic and Clinical Data

Data were collected using a standardized case-report form. Educational attainment was classified as primary school or below, secondary school, high school, or college or above. Clinical variables included epilepsy duration, epilepsy type, drug-resistant epilepsy, seizure frequency during the preceding 6 months, prior epilepsy surgery, and current antiseizure medication (ASM) regimen. Epilepsy type and drug resistance were defined according to ILAE criteria [33,34]. Seizure frequency was categorized as <1 or ≥1 seizure per month, and ASM regimen as ASM-free, monotherapy, or polytherapy.

### 2.3. Apparatus and Stimuli

Eye position was recorded at 1000 Hz with an infrared eye tracker (EM-2000 series, Chengdu Jasmines BioTech Inc., Chengdu, China). Stimuli were presented on a 27-inch, 100-Hz LCD monitor (27B2H2, AOC; TPV Electronics (Fujian) Co., Ltd., Fuqing, China; 1920 × 1080 pixels) at a viewing distance of 57 cm. Nine-point calibration and validation preceded testing. On each trial, 2–7 distinct shapes were sampled from eight geometric forms (circle, square, triangle, five-pointed star, heart, diamond, crescent, and cross). Each shape subtended approximately 2° and occupied an equally spaced position on the circumference of an imaginary circle of 10° radius centered on the screen.

### 2.4. Task and Procedure

Participants identified the changed item between a sample array and a subsequent test array (Figure 1). Each trial began with a central fixation cross and was terminated if fixation was not acquired within 2000 ms. After fixation was maintained for 500 ms, a sample array of 2–7 shapes was presented for 0.7 s per item (1400–4900 ms) with unrestricted viewing.

After a 1000 ms retention interval, the test array appeared at the same locations with one item replaced by a shape that had not appeared in the sample array. A response was registered, with auditory confirmation, after gaze remained for 900 ms within a 4–radius area of interest centered on one item; these areas did not overlap at any set size. The test array remained visible for at most 0.7 s per item plus 2 s (3400–6900 ms), and the trial ended if no response was registered within this interval. Set size began at 2, increased by one after three consecutive correct responses, and decreased by one after a single incorrect response, within a range of 2–7. The task comprised a single session of 40 trials.

### 2.5. Outcome Measures

#### 2.5.1. Task-Performance Measures

At each available set size, accuracy was the proportion of scorable trials in which the gaze-triggered response was registered within the area of interest of the changed item. Response time was measured from test-array onset to the onset of the 900 ms gaze period that registered the response; the 900 ms period itself was excluded. Final gaze-to-target distance was the Euclidean distance, in degrees of visual angle, between the final response-related gaze position and the center of the changed item. These three measures were treated as complementary task-performance measures, with no primary–secondary hierarchy assigned among them.

#### 2.5.2. Mean Set Size and K Estimates

Mean set size was each participant’s arithmetic mean across available set sizes. Because every participant began at set size 2 with a contiguous range, it equals (2 + highest set size reached)/2 and takes one of six values between 2.0 and 4.5. Mean set size was treated as a capacity-related measure rather than a continuous item-capacity estimate. Under the random-guessing change-localization formulation of Zhao et al. [16], the changed item is assumed to be represented in memory with probability K/N and, when represented, to be identified correctly. If the changed item is not represented, the participant is assumed to guess uniformly among the N locations, with a 1/N probability of a correct response. Expected accuracy is A_N = K/N + (1 − K/N)(1/N). Solving for K gives K = (N^2^A_N − N)/(N − 1). K at set size 4 (K4) was calculated as K4 = (16 × A4 − 4)/3, where A4 denotes accuracy at set size 4, using the change-localization formulation of Zhao et al. [16]. K4 was treated as an item-based, task-derived capacity estimate; because it is a deterministic, chance-corrected transformation of accuracy at set size 4, it was not treated as evidence independent of accuracy. K4 was used for group-level comparisons and was not used to classify individual participants. Negative values were retained in the primary analysis; sensitivity analyses floored negative K4 values at zero and examined K at set size 5, K5 = (25 × A5 − 5)/4.

#### 2.5.3. Eye-Movement Measures

Four exploratory measures were derived for each array. Saccades were identified using a velocity-based detection procedure. Candidate saccades were identified when absolute velocity exceeded 15°/s for more than 10 ms. For each candidate event, a segment-specific velocity threshold based on the presaccadic mean ± 2.58 SD was used to refine saccade onset and offset. Candidate detection did not use an amplitude or acceleration criterion; events with an amplitude below 1° were subsequently excluded from analysis [35]. Fixation intervals were defined as valid periods between detected saccades within the array period. For the test array, the final interval ended at response confirmation. Intervals lasting at least 40 ms were retained. No dispersion-based spatial threshold was used to identify fixation events.

Fixation count was the number of valid fixation intervals within the array period, and mean fixation duration was the arithmetic mean of the same intervals. Sample-array fixation rate was fixation count divided by sample-array duration. For trials with a registered response, test-array eye-movement measures were derived over the whole test-array period, from test-array onset through response confirmation, which included the 900 ms response-registration period and the terminal fixation interval. Because test-array duration depended on response timing, test-array fixation count was modeled with centered response time as a covariate rather than converted to a rate.

The 4–radius area of interest and the 900 ms sustained-gaze requirement defined response registration and were not used to identify fixation events. Convex hull area was the area (deg^2^) of the convex hull formed by fixations in that array. Percentage of stimuli fixated was the number of distinct stimuli receiving at least one fixation divided by the number presented, multiplied by 100.

### 2.6. Data Preprocessing

Participants were excluded when they were unable to complete the protocol or when their recordings were not suitable for reliable session-level eye-tracking analysis. A recording was considered unsuitable when acceptable nine-point calibration and validation could not be obtained after repeated attempts, when tracking was persistently lost, when a technical recording failure occurred, or when head or body position changed markedly during testing. No task-performance metric, including accuracy, response time, or the highest set size reached, was used as a participant-level exclusion criterion.

Within retained sessions, missing segments shorter than 50 ms were interpolated. The remaining invalid gaze samples, including those lost during blinks, were excluded from eye-movement coding within the affected task segment. An array segment was treated as invalid when continuous invalid gaze exceeded 200 ms or when invalid or missing samples exceeded 30% of that array period; either condition was sufficient. Trial inclusion was outcome-specific and required usable data from the relevant sample- or test-array segment; trials without a reliably determined gaze-triggered response were excluded from response-dependent outcomes.

### 2.7. Statistical Analysis

Analyses used Python 3.9.6 (statsmodels 0.14.5; SciPy 1.13.1) and MedCalc 20 (MedCalc Software Ltd., Ostend, Belgium). Age was summarized as median (interquartile range) and compared using the Mann–Whitney U test. Sex and four-level educational attainment were compared using chi-square tests, with Yates correction for sex; college-or-above education was compared using Fisher’s exact test.

For outcomes measured across set sizes, observed values were summarized as group means ± SD of each participant’s average across available set sizes; mean set size and K4 were summarized directly at the participant level. Cohen’s *d* was calculated from unrounded participant-level values using the pooled SD and reported as an absolute magnitude. Group was coded as controls = 0 and epilepsy = 1, and set size was centered at 4. The group coefficient estimated the epilepsy–control difference at set size 4. The set-size coefficient estimated the slope in controls, and the interaction estimated the between-group difference in slopes. All tests were two-sided.

#### 2.7.1. Models

Accuracy, response time, final gaze-to-target distance, and the eight eye-movement measures were analyzed using linear mixed-effect models with a participant random intercept: Outcome~Group × (Size − 4) + (1 | participant). Because set size was determined by the staircase procedure, each participant contributed observations only at the set sizes reached. Models were fitted by restricted maximum likelihood to all available participant-by-set-size observations, with Wald tests and 95% confidence intervals; the test-array fixation-count model additionally included centered response time. Mean set size and K4 were analyzed by ordinary least-squares regression with classical standard errors, with HC3 robust standard errors used in sensitivity analyses. Principal group-comparison models were also refitted with four-level educational attainment as a categorical covariate to assess robustness to residual imbalance in educational attainment within the analytic sample.

#### 2.7.2. Multiplicity and Sensitivity Analyses

Benjamini–Hochberg correction was applied separately to the following five families: task-performance group effects (three tests), task-performance interactions (three), mean set size and K4 (two), eye-movement group effects (eight), and eye-movement interactions (eight). The families reflected distinct inferential questions defined by outcome domain (task performance, capacity-related measures, or eye movements) and, for repeated-measure outcomes, by effect type (group effect or group × set-size interaction). Set-size slopes were not corrected. Statistical significance was defined as *q* < 0.05 when correction applied and *p* < 0.05 otherwise; unadjusted *p* values from sensitivity analyses were interpreted descriptively. Detected interactions were examined after excluding set size 7, set sizes 6–7, and set size 2.

#### 2.7.3. Overlap-Sample Analyses

Of the 355 participants included in the present study, 291 (198 with epilepsy and 93 controls) had also completed a saccadic task battery reported separately in a larger cohort of 890 participants [36]. The visual working memory task and the saccadic task battery were completed during the same testing session. The previous report examined prosaccade, antisaccade, memory-guided saccade, and double-step saccade outcomes and did not include the present visual working memory task or any of the capacity-related, task-performance, or array-level eye-movement outcomes reported here. Two previously reported prosaccade measures were used in the present study only as covariates in sensitivity analyses.

In this overlap subset, each model was refitted without covariates, with educational attainment, and, where applicable, with educational attainment plus the corresponding saccadic measure. Prosaccade latency was added to the response-time model and prosaccade spatial error to the final gaze-to-target-distance model; no saccadic covariate was specified for mean set size, K4, or accuracy. Because this subset was nested within the main sample, these analyses were treated as sensitivity analyses, and uncorrected *p* values were interpreted descriptively.

#### 2.7.4. Subgroup Analyses

Epilepsy-type analyses compared controls, focal epilepsy, and generalized epilepsy; participants with unknown epilepsy type were excluded because their epilepsy could not be classified as focal or generalized. The primary models were refitted with a three-level group variable, and pairwise contrasts were estimated from each model. Benjamini–Hochberg correction was applied separately within each outcome across the three pairwise contrasts. Drug-resistant status was examined within focal epilepsy, and the five drug-resistance comparisons constituted a separate Benjamini–Hochberg family. Subgroup analyses were exploratory and were not adjusted for educational attainment.

#### 2.7.5. Additional Analyses

Three additional analyses are reported in Supplementary Table S5. First, as a sensitivity analysis, the five base-model group effects for the core outcomes (accuracy, response time, final gaze-to-target distance, mean set size, and K4) were treated as a single Benjamini–Hochberg family. Second, as a restricted-range sensitivity analysis, the three task-performance models were refitted to set sizes 2–6; the resulting group and group × set-size *p* values were interpreted descriptively without additional multiplicity correction. Third, associations of clinical factors with the five core outcomes were examined within the epilepsy group. Seizure frequency, epilepsy duration in 5-year increments, drug-resistant status, and current ASM regimen were each entered in a separate model. Repeated-measure outcomes were analyzed with mixed-effect models including a participant random intercept and centered set size, without a clinical factor × set-size interaction, and mean set size and K4 were analyzed by ordinary least squares. Current ASM regimen was tested with a two-degree-of-freedom omnibus Wald test. These 20 tests formed one Benjamini–Hochberg family. The clinical-factor analyses were exploratory, used available cases without imputation, and were not adjusted for educational attainment.

## 3. Results

### 3.1. Participants and Clinical Characteristics

Of 388 participants assessed for eligibility (248 with epilepsy, 140 controls), 20 were excluded before testing (15 with epilepsy, five controls) because they did not meet the eligibility criteria or withdrew consent. A further 13 (11 with epilepsy, two controls) were excluded after testing because they were unable to complete the protocol or their eye-tracking recordings were not of sufficient quality. The analytic sample comprised 355 participants: 222 with epilepsy and 133 controls.

Demographic characteristics of the two groups and clinical characteristics of the epilepsy group are shown in Table 1. The groups did not differ in sex distribution, age, or the overall four-level distribution of educational attainment. The proportion of participants with college-level education or above was lower in the epilepsy group (Table 1).

### 3.2. Capacity-Related Measures and Task Performance Across Set Sizes

Group means at each set size are shown in Figure 2. In the two groups, accuracy decreased and response time and final gaze-to-target distance increased as set size increased. Across most set sizes, accuracy was lower in the epilepsy group, and response time and final gaze-to-target distance were higher. The separation between the groups did not widen visibly as set size increased. At the upper set sizes, group means were based on progressively fewer participants and had correspondingly wide confidence intervals. At set size 7, where the fewest participants contributed, the response-time means did not follow the ordering seen at the lower set sizes (Figure 2B).

At set size 4, the epilepsy group had lower accuracy, longer response time, and greater final gaze-to-target distance than controls. All three differences persisted after adjustment for educational attainment, while none of the group × set-size interactions were statistically significant. Model estimates, control-group set-size slopes, and effect-size magnitudes are reported in Table 2.

When the five core base-model group effects were corrected as a single Benjamini–Hochberg family, all five remained statistically significant (all *q* ≤ 0.003; Supplementary Table S5, Panel A). Restricting the three task-performance models to set sizes 2–6 left the group differences in the same direction and larger in absolute magnitude, while the group × set-size terms continued to provide no clear evidence of differential slopes (Supplementary Table S5, Panel B). The restricted-range *p* values were unadjusted and interpreted descriptively.

Mean set size and K4 were lower in the epilepsy group than in controls, and these estimates persisted after adjustment for educational attainment (Table 3). HC3 robust standard errors were examined as a sensitivity analysis of variance estimation. Flooring negative K4 values at zero left the K4 group difference in the same direction, and the K estimate at set size 5 was also directionally concordant (Supplementary Table S1).

In the 291-participant overlap subset, all five estimates were in the same direction as in the full sample, before and after adjustment for educational attainment. Further adjustment for prosaccade latency attenuated the response-time estimate, and further adjustment for prosaccade spatial error attenuated the final gaze-to-target distance estimate; neither changed direction. These were sensitivity analyses in a nested subset, and their *p* values were unadjusted (Supplementary Table S2).

### 3.3. Analyses by Epilepsy Type and Drug-Resistant Status

Participants with epilepsy of unknown type were excluded from the epilepsy-type analyses (Table 1), and these exploratory subgroup analyses were not adjusted for educational attainment.

Compared with controls, participants with focal epilepsy had a lower mean set size (β = −0.263; 95% CI, −0.391 to −0.134) and lower K4 (−0.297; −0.540 to −0.054), lower accuracy, longer response time, and greater final gaze-to-target distance (all *q* < 0.05). Participants with generalized epilepsy showed the same pattern for mean set size (−0.243; −0.435 to −0.052), K4 (−0.653; −1.014 to −0.292), accuracy, and final gaze-to-target distance (all *q* < 0.05); the response-time estimate was positive but imprecise (59.96 ms; −23.51 to 143.43) and did not survive correction.

Estimates relative to controls were directionally similar in focal and generalized epilepsy. In these unadjusted exploratory analyses, none of the direct focal-versus-generalized contrasts survived Benjamini–Hochberg correction, and none of the five comparisons between participants with and without drug-resistant epilepsy within focal epilepsy survived correction. Full estimates are reported in Supplementary Table S3.

Within the epilepsy group, none of the 20 exploratory associations of seizure frequency, epilepsy duration, drug-resistant status, or current antiseizure medication regimen with the five core outcomes survived Benjamini–Hochberg correction (Supplementary Table S5, Panel C).

### 3.4. Eye-Movement Measures During the Sample and Test Arrays

At set size 4, none of the eight group contrasts for the eye-movement measures survived Benjamini–Hochberg correction. The smallest unadjusted *p* values were obtained for sample-array mean fixation duration and sample-array fixation rate (Table 4).

One group × set-size interaction survived correction: the percentage of test-array stimuli fixated decreased more steeply with set size in the epilepsy group. The corresponding group contrast at set size 4 was not significant (Table 4). In restricted set-size ranges, the interaction estimate remained negative but became less precise; these *p* values were unadjusted (Supplementary Table S4).

## 4. Discussion

This study identified consistently poorer visual working memory task performance in adults with epilepsy than in healthy controls. The difference was expressed across capacity-related, temporal, and spatial response measures. Its magnitude did not change detectably across the observed set-size range. No broad corrected group differences were detected in the selected array-level eye-movement summaries. Using a graded-load, gaze-triggered change-localization task, the study provides a more specific account of how visual working memory performance differs across memory load. It also locates that difference within response timing, spatial response precision, and concurrent gaze behavior.

Working-memory impairment has been reported in pediatric epilepsy, genetic generalized epilepsy, and adult cohorts [10,11,12,13]. Previous eye-tracking studies have identified memory, attention, and oculomotor abnormalities in syndrome-specific epilepsy cohorts [29,37,38]. Earlier work has largely established whether these domains are affected; the present study asks how visual working memory performance differences are expressed across graded memory load. It brings capacity estimation, load dependence, response time, spatial response precision, and task-embedded gaze into the same experimental framework. The present group differences were small to moderate in magnitude (Table 2 and Table 3). Direct numerical comparison of effect magnitude across studies is inappropriate because studies differ in age range, task structure, response modality, and outcome definition.

Xiao et al. [29] and Zhang et al. [30] examined mesial temporal and frontal lobe epilepsy, respectively, in syndrome-specific cohorts. Their short-term memory games progressed from one to four targets, with mouse selection among 12 candidate items during retrieval and area-of-interest gaze measures recorded during encoding and retrieval. Eye movements were recorded alongside the behavioral response rather than serving as the response itself. Each study administered concurrent standardized memory measures and reported associations between memory-game or eye-tracking measures and memory-scale scores. In each study, the epilepsy group took longer to complete the memory game than controls. This direction is consistent with the longer response time observed here, but the endpoints are not equivalent. Completion time in the earlier paradigms included retrieval and manual selection of multiple targets, whereas response time in the present task ended at the onset of gaze-based response registration in a change-localization trial. The present study examined a larger cohort that included focal and generalized epilepsy and used an adaptive staircase across set sizes 2–7, without a concurrent standardized memory battery. A single accuracy score cannot distinguish a load-specific breakdown from a general shift in task efficiency. It also cannot show whether poorer performance is accompanied by broader changes in visual sampling.

The set-size analysis helps distinguish these possibilities. A deficit restricted to high loads would predict increasing group separation as set size rises. The observed pattern was more consistent with a broadly lower level of performance across the task’s operating range than with a discrete high-load breakdown. This interpretation is bounded by the staircase design. Participants contributed data only at loads they reached, fewer observations were available at upper set sizes, and the models tested linear rather than nonlinear interaction. Response-time means at set size 7, the most sparsely sampled load, did not follow the ordering seen at the lower set sizes. Restricting the task-performance models to set sizes 2–6 left the three group differences in the same direction and larger in absolute magnitude, while the group × set-size terms continued to provide no clear evidence of differential slopes. Task disengagement, rushed responding, and cognitive overload were not measured directly, so this descriptive pattern cannot be attributed to any of these mechanisms. The nonsignificant interactions indicate no detected effect modification across the observed range; they do not establish equivalent responses to increasing load [39].

The capacity-related measures should be interpreted as different representations of the same task system rather than independent demonstrations of separate deficits. K4 is a deterministic, chance-corrected transformation of accuracy at set size 4 [16,40]. Mean set size reflects the range reached under a staircase governed by response correctness [41]. It takes one of six discrete values determined by the highest set size reached under the staircase. Because the task was administered once, test–retest reliability of this measure was not assessed, limiting its interpretation for individual-level or longitudinal use. Their convergence with accuracy strengthens the description of the performance difference without multiplying the evidence. K4 remains useful because it expresses fixed-load performance in item units. The control estimate was of the same order as values reported in healthy adults using change-detection and change-localization paradigms [14,15,16,17]. This consistency supports the task’s interpretability but does not establish a normative threshold. Accuracy, response time, and final gaze-to-target distance are complementary response-level measures rather than isolated cognitive processes [42,43]. Lower accuracy together with longer response time is not the pattern expected from a simple speed–accuracy trade-off [44]. These measures provide quantitative group-level characterization of visual working memory performance; their value for identifying impairment in individual patients was not evaluated.

Adjustment for educational attainment did not materially alter the principal pattern, making educational imbalance alone a less likely explanation for the group differences. Partial attenuation after adjustment for the corresponding prosaccade measures suggests that response timing and spatial response precision share some variation with basic oculomotor performance. This relationship is informative because the gaze-triggered task combines cognitive and oculomotor demands, but it neither establishes independence from oculomotor function nor identifies mediation. The gaze-triggered response also has methodological value because it converts spatial selection into an explicit quantitative endpoint. In conventional categorical response paradigms, performance is typically represented by whether the correct item was selected. Gaze-triggered registration additionally provides a continuous spatial endpoint relative to the target. Final gaze-to-target distance is partly coupled to response correctness [40,45]. It also arises from a multistage process involving test-array inspection, memory comparison, visual search, decision formation, and response selection [26,42]. It should be interpreted as a spatial property of the memory-dependent response, not as an independent measure of basic saccadic precision [26,46]. Eye tracking adds temporal and spatial response information within the same task rather than replacing established neuropsychological assessment.

The array-level eye-movement findings further constrain interpretation. None of the selected sample- or test-array summaries showed a corrected group difference at set size 4. Test-array fixation count was descriptively higher and mean fixation duration descriptively shorter in PWE than in HC, but neither pattern was supported by the corrected group contrasts. These test-array summaries included the terminal response-related fixation during the 900 ms response-registration period and combined pre-response visual sampling with response registration rather than isolating visual search. The descriptive pattern should not be interpreted as evidence of fragmented visual search, increased distractibility, or compensation. The principal performance differences were not accompanied by a broad shift in these global measures of visual sampling. This does not establish equivalent eye movements. More specific abnormalities may still occur in fixation sequence, item prioritization, revisits, transitions, or information acquired per fixation [22,24,37].

Dedicated saccadic paradigms have detected abnormalities of initiation, inhibition, and spatial control in focal epilepsy [46] and in our previously reported larger cohort [36]. The contrast is compatible with task specificity. Oculomotor abnormalities may be more readily detected when eye-movement control is directly probed, whereas broad gaze summaries embedded within a memory task may be less sensitive. This is consistent with evidence that different eye-movement paradigms recruit different combinations of cognitive and oculomotor processes [47]. The single corrected interaction for the percentage of test-array stimuli fixated was less precise in restricted-range analyses and should remain hypothesis-generating.

Estimates relative to controls were directionally similar in focal and generalized epilepsy, consistent with reports that cognitive difficulties can span major epilepsy types [13,48,49]. The similar direction of estimates suggests that the observed group-level pattern may not be confined to one major epilepsy type. Direct focal-versus-generalized contrasts were imprecise and do not establish equivalence. The same caution applies to drug-resistant status. Treatment resistance does not directly index seizure burden, structural pathology, interictal activity, medication exposure, or network integrity. The absence of corrected differences by this classification should not be interpreted as absence of clinically relevant heterogeneity. The exploratory clinical-factor findings do not exclude contributions from seizure frequency, epilepsy duration, drug-resistant status, or current ASM regimen to task performance. The categorical ASM analysis did not evaluate individual drugs, doses, or cumulative exposure. These within-epilepsy analyses cannot determine whether treatment or other clinical characteristics account for the epilepsy–control differences.

### Limitations

Several limitations define the scope of inference. The study was cross-sectional and single-center, and residual confounding by education and clinical factors remains possible. Antiseizure medication exposure was available only as broad regimen categories. No concurrent standardized neuropsychological battery was administered, so convergent validity and incremental clinical value relative to established neuropsychological assessment could not be assessed. No non-memory control condition was included, so the observed epilepsy–control differences cannot be attributed specifically to memory storage rather than to contributions from encoding, visual search, decision, or response-selection processes. Because the replacement shape was globally novel, the task cannot determine whether participants relied on position-specific comparison, global novelty detection, or a combination of these strategies. The staircase procedure yielded progressively fewer observations at higher set sizes, limiting tests of nonlinear load effects. Eye-movement measures were global summaries and may miss sequence- or item-level abnormalities. The overlap analyses were sensitivity analyses within the main cohort rather than independent replication, and subgroup estimates were less precise. Participants with unknown epilepsy type were excluded only from the epilepsy-type comparisons, so those estimates apply to participants classified as having focal or generalized epilepsy and do not encompass the full clinical heterogeneity of the overall epilepsy cohort. Test–retest reliability of the task-derived measures was not assessed, limiting their interpretation for individual-level or repeated assessment. Future work should establish test–retest reliability, compare the task with standardized neuropsychological measures and everyday cognitive function, and assess sensitivity to longitudinal clinical change.

## 5. Conclusions

Adults with epilepsy showed lower task-estimated visual working memory capacity and poorer task performance than healthy controls, without broad corrected group differences in the selected array-level eye-movement measures. The gaze-triggered task provides capacity-related measures together with response timing and spatial response precision within a single experimental framework.

## Figures and Tables

**Figure 1:**
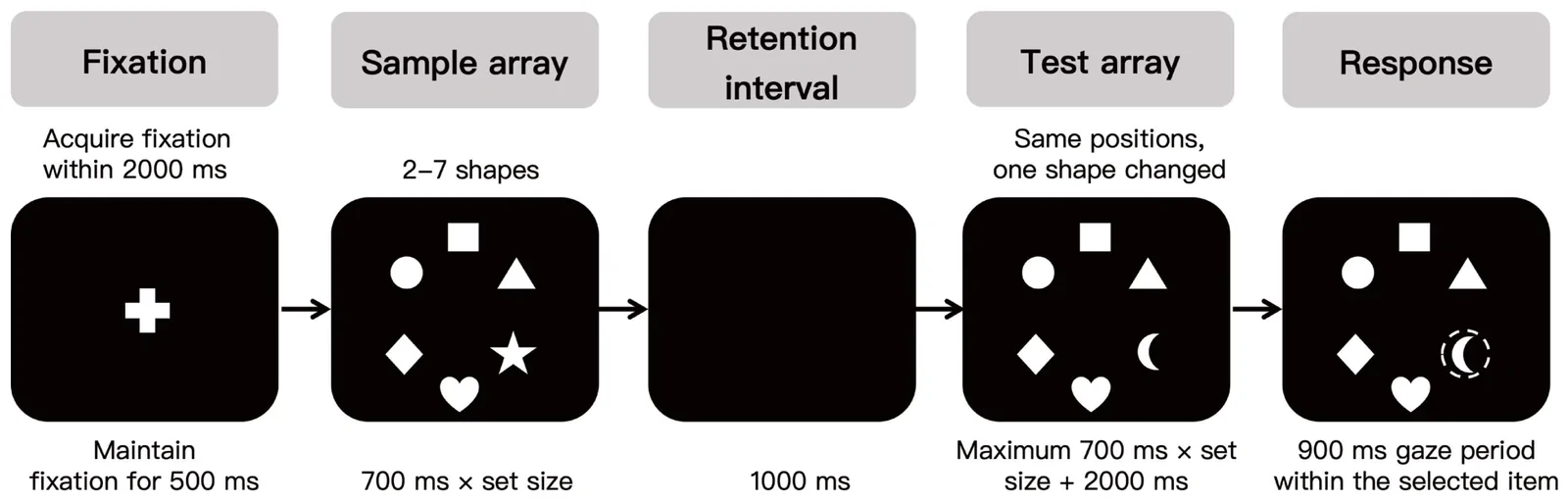
**Trial sequence of the visual working memory task.** Panels show successive stages of one trial. The test array and response panels are two moments within the same continuously visible display rather than two separate displays. The dashed circle marks the area of interest of the selected item and was not visible to participants. The example illustrates a set size of 6. The task and its timing are described in Section 2.4.

**Figure 2:**
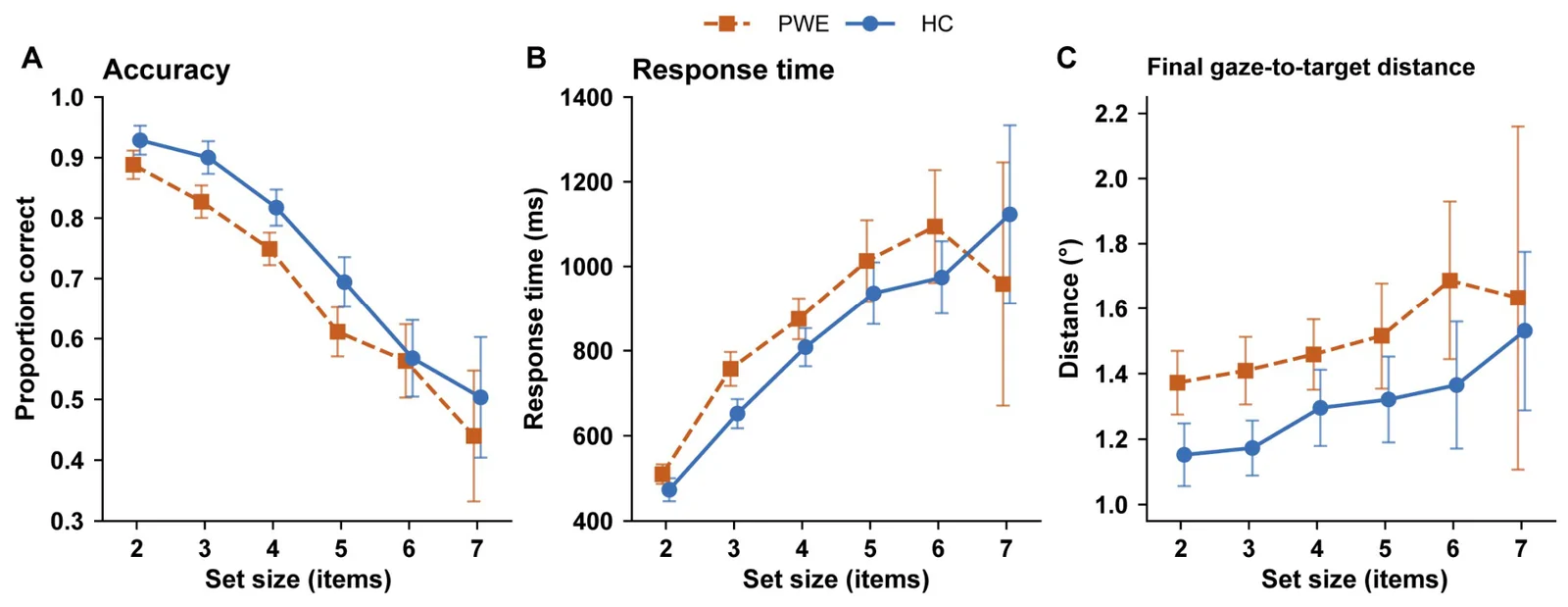
**Task-performance outcomes across set sizes in people with epilepsy (PWE) and healthy controls (HCs).** Panels show (**A**) accuracy, expressed as proportion correct; (**B**) response time; and (**C**) final gaze-to-target distance. Points represent observed group means, and error bars represent 95% confidence intervals. PWEs are orange squares with dashed lines; HCs are blue circles with solid lines. Sample sizes decrease at the upper set sizes and differ between panel (**A**) and panels (**B**,**C**). PWE/HC sample sizes at set sizes 2–7 were 222/133, 221/133, 209/130, 179/124, 113/91, and 47/49 for accuracy, and 222/133, 219/132, 204/130, 160/119, 93/77, and 30/37 for response time and final gaze-to-target distance. Model-based results are reported in Table 2.

**Table 1:** Demographic characteristics of the study sample and clinical characteristics of participants with epilepsy.

Variables	PWE Group (*n* = 222)	HC Group (*n* = 133)	*p* Value
**Demographic characteristics**			
Sex, *n* (%)			0.202
Male	95 (42.8)	47 (35.3)	
Female	127 (57.2)	86 (64.7)	
Age, years, median (IQR)	29 (24–40)	27 (25–35)	0.341
Education level, *n* (%)			0.064
Primary school or below	13 (5.9)	3 (2.3)	
Secondary school	6 (2.7)	1 (0.8)	
High school	20 (9.0)	6 (4.5)	
College or above	183 (82.4)	123 (92.5)	0.010 ‡
**Clinical characteristics of participants with epilepsy**			
Drug-resistant epilepsy, *n* (%)			
Yes	52 (23.4)	—	
No	170 (76.6)	—	
Epilepsy duration, years, median (IQR)	11 (5–16)	—	
Prior epilepsy surgery, *n* (%)			
Yes	11 (5.0)	—	
No	211 (95.0)	—	
Epilepsy type, *n* (%)			
Generalized	42 (18.9)	—	
Focal	151 (68.0)	—	
Unknown	29 (13.1)	—	
Seizure frequency, *n* (%)			
<1 seizure/month	136 (61.3)	—	
≥1 seizure/month	86 (38.7)	—	
Current antiseizure medication regimen, *n* (%)			
Monotherapy	102 (45.9)	—	
Polytherapy	102 (45.9)	—	
ASM-free	18 (8.1)	—	

**Note.** Data are *n* (%) for categorical variables and median (interquartile range) for continuous variables. Percentages use the group total as the denominator. *p* values compare the two groups; no correction for multiple comparisons was applied within this table. ‡ College-or-above versus below-college-level educational attainment, reported separately from the four-level comparison above. Clinical characteristics are reported for the epilepsy group only, as these variables do not apply to controls. Statistical methods are described in Section 2.7. Abbreviations: PWE, people with epilepsy; HC, healthy controls; IQR, interquartile range; ASM, antiseizure medication; —, not applicable.

**Table 2:** Group comparisons for task-performance outcomes across set sizes.

	Accuracy	Response Time, ms	Final Gaze-to-Target Distance, Deg
**Observed values**			
PWE, mean ± SD	0.71 ± 0.15	811.83 ± 266.17	1.54 ± 0.68
HC, mean ± SD	0.76 ± 0.12	764.33 ± 186.87	1.28 ± 0.48
Cohen’s *d*, magnitude	0.33	0.20	0.41
**PWE − HC at set size 4, base model**			
β	−0.067	79.50	0.253
95% CI	−0.102 to −0.033	27.57 to 131.43	0.128 to 0.379
*p*	<0.001	0.003	<0.001
*q*	<0.001	0.003	<0.001
**PWE − HC at set size 4, education-adjusted model**			
β	−0.050	72.93	0.226
95% CI	−0.082 to −0.017	20.44 to 125.43	0.100 to 0.352
*p*	0.003	0.006	<0.001
**Group × set size**			
β	−0.0034	9.301	0.0197
95% CI	−0.0166 to 0.0097	−15.628 to 34.230	−0.0276 to 0.0670
*p*	0.608	0.465	0.415
*q*	0.608	0.608	0.608
**Set-size slopes in the HC group**			
β	−0.104	133.60	0.084
95% CI	−0.113 to −0.094	114.95 to 152.25	0.048 to 0.119
*p*	<0.001	<0.001	<0.001

**Note.** Observed values are group means ± SD of each participant’s average across available set sizes (PWE *n* = 222, HC *n* = 133); they are not set-size-specific model estimates. β values represent PWE minus HC: negative values indicate lower accuracy, whereas positive values indicate longer response time or greater distance in PWE. The group × set-size β is the between-group difference in set-size slopes. Accuracy is on a 0–1 scale, so β = −0.067 corresponds to 6.7 percentage points. Cohen’s *d* is an absolute descriptive magnitude calculated from unrounded participant-level values, not a standardized model estimate. Models, covariates, and multiplicity correction are described in Section 2.7. Abbreviations: PWE, people with epilepsy; HCs, healthy controls; CI, confidence interval; SD, standard deviation.

**Table 3:** Group comparisons for mean set size and K at set size 4.

	Mean Set Size	K at Set Size 4 (K4), Items
**Observed values**		
PWE, mean ± SD	3.73 ± 0.58	2.66 ± 1.05
HC, mean ± SD	3.98 ± 0.50	3.03 ± 0.92
*n* (PWE/HC)	222/133	209/130
Cohen’s *d*, magnitude	0.45	0.36
**PWE − HC, base model**		
β	−0.249	−0.363
95% CI	−0.368 to −0.131	−0.584 to −0.142
*p*	<0.001	0.001
*q*	<0.001	0.001
**PWE − HC, education-adjusted model**		
β	−0.201	−0.321
95% CI	−0.313 to −0.088	−0.544 to −0.098
*p*	<0.001	0.005

**Note.** K4 is a chance-corrected transformation of accuracy at set size 4 and is not independent confirmatory evidence from accuracy. β values represent PWE minus HC; negative values indicate lower values in PWE. K4 is expressed in items. Cohen’s *d* is an absolute descriptive magnitude, not a standardized form of the model estimate. The two measures, the models and multiplicity correction are described in Section 2.5 and Section 2.7. Abbreviations: PWE, people with epilepsy; HCs, healthy controls; CI, confidence interval; SD, standard deviation.

**Table 4:** Group comparisons and group × set-size interactions for eye-movement measures during the sample and test arrays.

Sample Array				
	**Fixation rate, n/s**	**Mean fixation duration, ms**	**Convex hull area, deg^2^**	**Stimuli fixated, %**
PWE, mean ± SD	3.02 ± 0.55	294.54 ± 109.56	105.88 ± 43.35	81.12 ± 20.53
HC, mean ± SD	2.94 ± 0.59	319.36 ± 161.00	121.72 ± 42.43	81.92 ± 21.68
Base model β	0.108	−30.03	−3.377	−0.848
95% CI	−0.015 to 0.231	−59.00 to −1.06	−11.858 to 5.105	−5.384 to 3.687
*p*	0.084	0.042	0.435	0.714
*q*	0.275	0.275	0.870	0.937
Education-adjusted β	0.103	−28.35	−3.263	−1.024
95% CI	−0.021 to 0.227	−57.59 to 0.90	−11.838 to 5.311	−5.617 to 3.569
*p*	0.102	0.057	0.456	0.662
Group × set size β	0.015	1.027	−0.407	−0.616
95% CI	−0.009 to 0.040	−6.840 to 8.895	−3.258 to 2.444	−1.502 to 0.270
*p*	0.222	0.798	0.780	0.173
*q*	0.444	0.798	0.798	0.444
**Test array**				
	**Fixation count**	**Mean fixation duration, ms**	**Convex hull area, deg^2^**	**Stimuli fixated, %**
PWE, mean ± SD	3.67 ± 1.04	362.18 ± 97.47	54.28 ± 26.98	56.62 ± 14.80
HC, mean ± SD	3.54 ± 0.82	376.97 ± 107.80	60.54 ± 24.97	53.32 ± 14.03
Base model β	−0.005	−18.46	−0.645	0.753
95% CI	−0.129 to 0.119	−40.66 to 3.74	−6.205 to 4.915	−1.923 to 3.430
*p*	0.939	0.103	0.820	0.581
*q*	0.939	0.275	0.937	0.930
Education-adjusted β	−0.021	−14.54	−1.069	0.257
95% CI	−0.146 to 0.104	−36.87 to 7.79	−6.699 to 4.560	−2.442 to 2.956
*p*	0.743	0.202	0.710	0.852
Group × set size β	0.022	3.221	−0.712	−1.545
95% CI	−0.012 to 0.056	−3.962 to 10.404	−3.640 to 2.216	−2.465 to −0.625
*p*	0.209	0.379	0.634	0.001
*q*	0.444	0.607	0.798	0.008

**Note.** Observed values are participant-level averages across available set sizes (PWE *n* = 222, HC *n* = 133), not set-size-specific estimates. β values represent PWE minus HC. Base and education-adjusted β values are group differences at set size 4; group × set-size β values are differences in set-size slopes. Sample-array fixation rate is reported as n/s, whereas test-array fixation count is used because test-array duration depended on response timing (Section 2.5). Measure definitions, models, and multiplicity correction are described in Section 2.5 and Section 2.7. Abbreviations: PWE, people with epilepsy; HCs, healthy controls; CI, confidence interval; SD, standard deviation.

## Data Availability

The data presented in this study are available on request from the corresponding author. The data are not publicly available because they contain clinical information from which individual participants could potentially be identified.

## Supplementary Materials

The following supporting information can be downloaded at https://www.mdpi.com/article/10.3390/diagnostics16193156/s1, Table S1: Sensitivity analyses for mean set size and K estimates; Table S2: Analyses in the 291-participant overlap sample; Table S3: Analyses by epilepsy type and drug-resistant status; Table S4: Sensitivity analyses for the group × set-size interaction in the percentage of test-array stimuli fixated; Table S5: Additional sensitivity and exploratory analyses.
