# Supplementary material for: Lower Task-Estimated Visual Working Memory Capacity in Adults with Epilepsy: A Cross-Sectional Quantitative Assessment Using Eye Tracking
Source: Diagnostics (Basel). 2026 Sep 28;16(19):3156. doi: 10.3390/diagnostics16193156 (PMC13664567; doi:10.3390/diagnostics16193156)
Supplement: Supplementary file 1 [file diagnostics-16-03156-s001.zip › Supplementary Table S5.pdf]

Supplementary Table S5. Additional sensitivity and exploratory analyses.

Panel A. Joint Benjamini–Hochberg sensitivity analysis for the five core group-comparison outcomes

Outcome	β	95% CI	p	Joint BH-adjusted q
Accuracy	−0.067	−0.102 to −0.033	<0.001	<0.001
Response time, ms	79.50	27.57 to 131.43	0.003	0.003
Final gaze-to-target distance, deg	0.253	0.128 to 0.379	<0.001	<0.001
Mean set size	−0.249	−0.368 to −0.131	<0.001	<0.001
K at set size 4 (K4), items	−0.363	−0.584 to −0.142	0.001	0.002

Note. The five base-model group effects were additionally treated as a single Benjamini–Hochberg family; the corresponding family-specific q values from the principal analyses are reported in Tables 2 and 3. The correction used unrounded p values from the fitted models. β values represent PWE minus HC. For accuracy, response time, and final gaze-to-target distance, β represents the group difference at set size 4. Negative values indicate lower accuracy, mean set size, or K4 in PWE; positive values indicate longer response time or greater final gaze-to-target distance in PWE. Statistical methods are described in Section 2.7 of the main manuscript.

Panel B. Sensitivity analysis of task-performance outcomes restricted to set sizes 2–6

Outcome	PWE − HC β at set size 4	95% CI	p	Group × set size β	95% CI	p	n	N obs.
Accuracy	−0.068	−0.103 to −0.033	<0.001	−0.0040	−0.0180 to 0.0099	0.570	355	1555
Response time, ms	90.33	38.46 to 142.21	<0.001	20.81	−4.93 to 46.55	0.113	355	1489
Final gaze-to-target distance, deg	0.263	0.137 to 0.388	<0.001	0.0314	−0.0199 to 0.0827	0.230	355	1489

Note. β values represent PWE minus HC at set size 4; the group × set size β represents the between-group difference in set-size slopes. No multiplicity correction was applied to these sensitivity analyses. N obs. denotes participant-by-set-size observations. Statistical methods are described in Section 2.7 of the main manuscript.

Panel C. Exploratory associations of clinical factors with the five core outcomes in participants with epilepsy

Outcome	n	N obs.	β (95% CI)	Omnibus Wald χ^2 (df)	p	BH-adjusted q
**Seizure frequency (≥1 versus <1 seizure per month)**						
Accuracy	222	991	0.020 (−0.027 to 0.066)	—	0.405	0.811
Response time, ms	222	928	72.63 (−0.97 to 146.24)	—	0.053	0.531
Final gaze-to-target distance, deg	222	928	0.033 (−0.139 to 0.205)	—	0.709	0.811
Mean set size	222	—	−0.028 (−0.184 to 0.129)	—	0.729	0.811
K at set size 4 (K4), items	209	—	0.066 (−0.228 to 0.360)	—	0.661	0.811
**Epilepsy duration (per 5-year increase)**						
Accuracy	222	991	−0.004 (−0.020 to 0.012)	—	0.600	0.811
Response time, ms	222	928	7.43 (−17.90 to 32.76)	—	0.565	0.811
Final gaze-to-target distance, deg	222	928	−0.012 (−0.070 to 0.047)	—	0.692	0.811
Mean set size	222	—	−0.031 (−0.084 to 0.021)	—	0.235	0.785
K at set size 4 (K4), items	209	—	−0.034 (−0.133 to 0.064)	—	0.492	0.811
**Drug-resistant epilepsy (yes versus no)**						

Outcome	n	N obs.	β (95% CI)	Omnibus Wald χ^2 (df)	p	BH-adjusted q
Accuracy	222	991	−0.061 (−0.114 to −0.007)	—	0.026	0.529
Response time, ms	222	928	1.26 (−85.55 to 88.08)	—	0.977	0.977
Final gaze-to-target distance, deg	222	928	0.052 (−0.148 to 0.252)	—	0.609	0.811
Mean set size	222	—	−0.140 (−0.319 to 0.040)	—	0.126	0.631
K at set size 4 (K4), items	209	—	−0.096 (−0.443 to 0.251)	—	0.588	0.811
**Current antiseizure medication regimen (three levels)**						
Accuracy	222	991	—	0.184 (2)	0.912	0.960
Response time, ms	222	928	—	1.740 (2)	0.419	0.811
Final gaze-to-target distance, deg	222	928	—	3.643 (2)	0.162	0.647
Mean set size	222	—	—	0.695 (2)	0.706	0.811
K at set size 4 (K4), items	209	—	—	4.193 (2)	0.123	0.631

Note. Analyses were restricted to participants with epilepsy and were exploratory. Estimates represent associations across the observed set-size range. For current ASM regimen, p and q values are based on a two-degree-of-freedom omnibus Wald test, for which no single β is defined. The 20 tests constituted one Benjamini–Hochberg family, and interpretation was based on the adjusted q values. N obs. denotes participant-by-set-size observations for repeated-measures outcomes. Statistical methods are described in Section 2.7 of the main manuscript.

Abbreviations: PWE, people with epilepsy; HC, healthy controls; CI, confidence interval; K4, K at set size 4; ASM, antiseizure medication; BH, Benjamini–Hochberg.
