# Supplementary material for: Lower Task-Estimated Visual Working Memory Capacity in Adults with Epilepsy: A Cross-Sectional Quantitative Assessment Using Eye Tracking
Source: Diagnostics (Basel). 2026 Sep 28;16(19):3156. doi: 10.3390/diagnostics16193156 (PMC13664567; doi:10.3390/diagnostics16193156)
Supplement: Supplementary file 1 [file diagnostics-16-03156-s001.zip › Supplementary Table S1.pdf]

Supplementary Table S1. Sensitivity analyses for mean set size and K estimates

Analysis	Outcome	n	PWE − HC β (95% CI)	p
**Base model (HC3)**	Mean set size	355	−0.249 (−0.364 to −0.135)	<0.001
**Education-adjusted (HC3)**	Mean set size	355	−0.201 (−0.308 to −0.094)	<0.001
**Base model (HC3)**	K at set size 4 (K4), items	339	−0.363 (−0.577 to −0.149)	<0.001
**Education-adjusted (HC3)**	K at set size 4 (K4), items	339	−0.321 (−0.539 to −0.103)	0.004
**K4 floored at 0 — base OLS**	K4 (negative values set to 0)	339	−0.331 (−0.537 to −0.125)	0.002
**K4 floored at 0 — education-adjusted OLS**	K4 (negative values set to 0)	339	−0.286 (−0.493 to −0.079)	0.007
**K4 floored at 0 — base HC3**	K4 (negative values set to 0)	339	−0.331 (−0.536 to −0.126)	0.002
**K4 floored at 0 — education-adjusted HC3**	K4 (negative values set to 0)	339	−0.286 (−0.492 to −0.080)	0.007
**K5 — base OLS**	K at set size 5 (K5), items	303	−0.518 (−0.891 to −0.146)	0.007
**K5 — education-adjusted OLS**	K at set size 5 (K5), items	303	−0.464 (−0.839 to −0.089)	0.016
**K5 — base HC3**	K at set size 5 (K5), items	303	−0.518 (−0.879 to −0.157)	0.005
**K5 — education-adjusted HC3**	K at set size 5 (K5), items	303	−0.464 (−0.825 to −0.102)	0.012

Note. All models in this table are sensitivity analyses; "base" denotes models without adjustment for educational attainment, and HC3 denotes heteroskedasticity-consistent standard errors. Primary inference for mean set size and K4 in the main text used ordinary least-squares (OLS) models with classical standard errors. β values represent PWE minus HC; negative values indicate lower values in PWE. K4 was calculated as (16 × accuracy at set size 4 − 4)/3 and K5 as (25 × accuracy at set size 5 − 5)/4. In the floored sensitivity analysis, negative K4 values were set to 0 before model fitting. Negative participant-level K estimates occurred in 5 of 339 participants for K4 and in 25 of 303 participants for K5. Education-adjusted models included four-level educational attainment as a categorical factor. P values from these sensitivity analyses were not adjusted for multiple comparisons. All sensitivity analyses supported the same directional conclusions as the primary models.
