# Supplementary material for: Lower Task-Estimated Visual Working Memory Capacity in Adults with Epilepsy: A Cross-Sectional Quantitative Assessment Using Eye Tracking
Source: Diagnostics (Basel). 2026 Sep 28;16(19):3156. doi: 10.3390/diagnostics16193156 (PMC13664567; doi:10.3390/diagnostics16193156)
Supplement: Supplementary file 1 [file diagnostics-16-03156-s001.zip › Supplementary Table S2.pdf]

Supplementary Table S2. Analyses in the 291-participant overlap sample

Outcome	Model	Sample detail	PWE − HC β (95% CI)	p
**Mean set size**	355 — base OLS	PWE 222 / HC 133	−0.249 (−0.368 to −0.131)	<0.001
	291 — base OLS	PWE 198 / HC 93	−0.310 (−0.444 to −0.176)	<0.001
	291 — education-adjusted OLS	n = 291	−0.251 (−0.380 to −0.122)	<0.001
**K4, items**	355 — base OLS	PWE 209 / HC 130	−0.363 (−0.584 to −0.142)	0.001
	291 — base OLS	PWE 186 / HC 93	−0.359 (−0.613 to −0.106)	0.006
	291 — education-adjusted OLS	n = 279	−0.317 (−0.571 to −0.063)	0.015
**Accuracy**	355 — base LMM	PWE 222 / HC 133	−0.067 (−0.102 to −0.033)	<0.001
	291 — base LMM	PWE 198 / HC 93	−0.071 (−0.110 to −0.031)	<0.001
	291 — education-adjusted LMM	n = 291	−0.051 (−0.089 to −0.014)	0.007
**Response time, ms**	355 — base LMM	PWE 222 / HC 133	79.50 (27.57 to 131.43)	0.003
	291 — base LMM	PWE 198 / HC 93	85.85 (25.50 to 146.21)	0.005
	291 — education-adjusted LMM	n = 291	77.68 (16.69 to 138.66)	0.013
	291 — education + prosaccade latency	n = 291	65.07 (2.66 to 127.48)	0.041
**Final gaze-to-target distance, deg**	355 — base LMM	PWE 222 / HC 133	0.253 (0.128 to 0.379)	<0.001
	291 — base LMM	PWE 198 / HC 93	0.310 (0.164 to 0.457)	<0.001
	291 — education-adjusted LMM	n = 291	0.272 (0.126 to 0.417)	<0.001
	291 — education + prosaccade spatial error	n = 291	0.236 (0.090 to 0.382)	0.002

Note. The 291-participant overlap sample comprises participants with available data from both the current visual working memory task and the previously reported saccadic task battery. β values represent PWE minus HC; negative values indicate lower values in PWE, whereas positive values indicate higher values in PWE. Model sequence for each outcome: (i) full-sample (n = 355) base model; (ii) 291-participant base model; (iii) 291-participant education-adjusted model; and, where applicable, (iv) 291-participant education-adjusted model with the corresponding saccadic measure. Response time was additionally adjusted for prosaccade latency and final gaze-to-target distance for prosaccade spatial error; no saccadic covariate was included for mean set size, K4, or accuracy. Outcomes with a single value per participant (mean set size, K4) were analyzed by ordinary least squares; outcomes with one value per participant at each set size were analyzed with linear mixed-effects models, Outcome ~ Group × (Size − 4) + (1 | participant), consistent with the main text. For the mixed-effects models, the group estimate is the PWE − HC difference at set size 4. Within the overlap subset, K4 was available for 279 participants (186 PWE and 93 HC), because accuracy at set size 4 was not available for every participant. P values in this sensitivity analysis were unadjusted and were interpreted descriptively.
