# Supplementary material for: Lower Task-Estimated Visual Working Memory Capacity in Adults with Epilepsy: A Cross-Sectional Quantitative Assessment Using Eye Tracking
Source: Diagnostics (Basel). 2026 Sep 28;16(19):3156. doi: 10.3390/diagnostics16193156 (PMC13664567; doi:10.3390/diagnostics16193156)
Supplement: Supplementary file 1 [file diagnostics-16-03156-s001.zip › Supplementary Table S3.pdf]

Supplementary Table S3. Analyses by epilepsy type and drug-resistant status

Panel A. Epilepsy type comparisons (HC, focal epilepsy, generalized epilepsy)

Outcome	Contrast	n (HC / focal / generalized)	β (95% CI)	p	q
**Mean set size**	Focal vs HC	133 / 151 / 42	−0.263 (−0.391 to −0.134)	<0.001	<0.001
	Generalized vs HC	133 / 151 / 42	−0.243 (−0.435 to −0.052)	0.013	0.019
	Focal vs generalized	133 / 151 / 42	−0.020 (−0.208 to 0.169)	0.839	0.839
**K4, items**	Focal vs HC	130 / 141 / 40	−0.297 (−0.540 to −0.054)	0.017	0.025
	Generalized vs HC	130 / 141 / 40	−0.653 (−1.014 to −0.292)	<0.001	0.001
	Focal vs generalized	130 / 141 / 40	0.356 (−0.002 to 0.714)	0.051	0.051
**Accuracy**	Focal vs HC	133 / 151 / 42	−0.0725 (−0.1102 to −0.0348)	<0.001	<0.001
	Generalized vs HC	133 / 151 / 42	−0.0843 (−0.1407 to −0.0279)	0.003	0.005
	Focal vs generalized	133 / 151 / 42	0.0118 (−0.0441 to 0.0677)	0.680	0.680
**Response time, ms**	Focal vs HC	133 / 151 / 42	96.33 (40.67 to 151.98)	<0.001	0.002
	Generalized vs HC	133 / 151 / 42	59.96 (−23.51 to 143.43)	0.159	0.239
	Focal vs generalized	133 / 151 / 42	36.37 (−46.89 to 119.64)	0.392	0.392
**Final gaze-to-target distance, deg**	Focal vs HC	133 / 151 / 42	0.235 (0.098 to 0.373)	<0.001	0.002
	Generalized vs HC	133 / 151 / 42	0.303 (0.097 to 0.510)	0.004	0.006
	Focal vs generalized	133 / 151 / 42	−0.068 (−0.273 to 0.137)	0.516	0.516

Note (Panel A). Participants with epilepsy of unknown type were excluded. In each contrast, β values represent the first-named group minus the second-named group. Outcomes with a single value per participant were analyzed by ordinary least-squares models with a three-level epilepsy-type factor. Outcomes with one value per participant at each set size were analyzed with linear mixed-effects models including epilepsy type × (Size − 4) and a participant random intercept; reported β values are contrasts at set size 4. Pairwise contrasts were estimated from the fitted three-level model. The n column gives the numbers contributing to the fitted three-level model and is therefore identical for all three contrasts of a given outcome. Benjamini–Hochberg correction was applied separately within each outcome across its three pairwise contrasts (five separate families of three tests). These exploratory analyses were not adjusted for educational attainment.

Panel B. Drug-resistant versus non–drug-resistant status within focal epilepsy

Outcome	n (DRE / non-DRE)	DRE − non-DRE β (95% CI)	p	q
**Mean set size**	31 / 120	−0.153 (−0.387 to 0.080)	0.197	0.491
**K4, items**	27 / 114	−0.060 (−0.473 to 0.352)	0.772	0.772
**Accuracy**	31 / 120	−0.0643 (−0.1285 to −0.0001)	0.050	0.249
**Response time, ms**	31 / 120	52.90 (−58.06 to 163.86)	0.350	0.583
**Final gaze-to-target distance, deg**	31 / 120	0.091 (−0.160 to 0.342)	0.477	0.596

Note (Panel B). Analyses were restricted to focal epilepsy, using the same dataset and outcome definitions as the main text, including K4 = (16 × accuracy at set size 4 − 4)/3 and models centered at set size 4. β values represent participants with drug-resistant epilepsy (DRE) minus those without. Outcomes with a single value per participant were analyzed by ordinary least squares; outcomes with one value per participant at each set size were analyzed with linear mixed-effects models including DRE × (Size − 4). Benjamini–Hochberg correction was applied across the five outcome comparisons as a single family; no comparison remained statistically significant after correction. These exploratory analyses were not adjusted for educational attainment.
