# Supplementary material for: Lower Task-Estimated Visual Working Memory Capacity in Adults with Epilepsy: A Cross-Sectional Quantitative Assessment Using Eye Tracking
Source: Diagnostics (Basel). 2026 Sep 28;16(19):3156. doi: 10.3390/diagnostics16193156 (PMC13664567; doi:10.3390/diagnostics16193156)
Supplement: Supplementary file 1 [file diagnostics-16-03156-s001.zip › Supplementary Table S4.pdf]

Supplementary Table S4. Sensitivity analyses for the group × set-size interaction in the percentage of test-array stimuli fixated

Analysis set	Group × set-size β (95% CI)	p	HC set-size slope β (95% CI)	PWE set-size slope β (95% CI)
**Set sizes 2–7 (primary)**	−1.545 (−2.465 to −0.625)	0.001	−6.669 (−7.356 to −5.981)	−8.214 (−8.827 to −7.600)
**Excluding set size 7**	−1.128 (−2.131 to −0.125)	0.027	−7.528 (−8.292 to −6.764)	−8.656 (−9.306 to −8.005)
**Excluding set sizes 6–7**	−1.225 (−2.436 to −0.014)	0.047	−8.592 (−9.527 to −7.658)	−9.817 (−10.588 to −9.046)
**Excluding set size 2**	−1.281 (−2.490 to −0.073)	0.038	−4.538 (−5.431 to −3.646)	−5.820 (−6.637 to −5.003)

Note. Outcome: percentage of test-array stimuli fixated. Models: Outcome ~ Group × (Size − 4) + (1 | participant), fitted by restricted maximum likelihood. The group × set-size coefficient is the difference in set-size slopes (PWE − HC), in percentage points per one-item increase in set size. In the primary analysis (set sizes 2–7) this interaction was $\beta = -1.545$ (95% CI, −2.465 to −0.625), $p = 0.001$, $q = 0.008$ after Benjamini–Hochberg correction within the Table 4 interaction family. The HC and PWE set-size slopes are the group-specific set-size slopes estimated from the same model. The sensitivity analyses restricted the set-size range in alternative ways to evaluate the influence of the extreme set sizes. The interaction remained negative with $p < 0.05$ in all three restricted analyses, with attenuated magnitude and wider confidence intervals, as expected when the set-size levels contributing most to the difference in slopes are removed. P values for the restricted-range analyses were unadjusted and are interpreted descriptively.
